# Lumbar *Brucella* Spondylodiscitis with Extensive Vertebral Abscesses

**DOI:** 10.4269/ajtmh.24-0619

**Published:** 2025-04-01

**Authors:** Shutao Gao, Yukun Hu, Weibin Sheng

**Affiliations:** Department of Spine Surgery, Xinjiang Medical University Affiliated First Hospital, Urumqi, China

**Figure 1.**
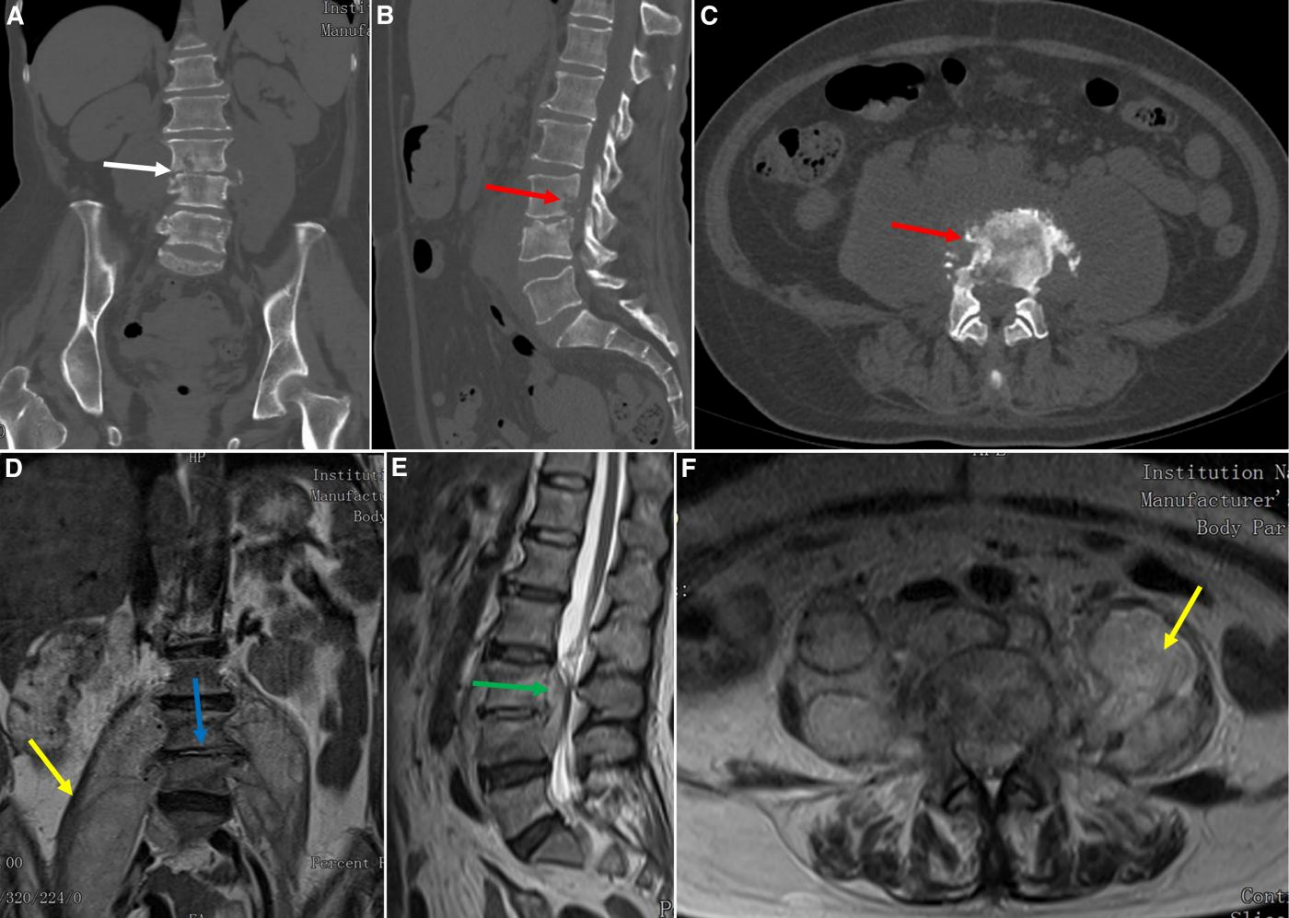
Preoperative computed tomography (CT) and magnetic resonance imaging (MRI) scans. (**A–C**) Preoperative coronal, sagittal, and axial CT images indicate a collapsed L3/4 intervertebral space (white arrow) and bony destruction of the L3 and L4 vertebrae (red arrow). (**D–F**) Preoperative coronal, sagittal, and axial MRI scans show L3/4 spondylodiscitis (blue arrow), a large psoas abscess (yellow arrow), and an epidural abscess (green arrow).

A 59-year-old woman was referred to the outpatient department, complaining of 2 months of low back pain and intermittent fever. She is a shepherd and lives in a village in the Aksu Prefecture of Xinjiang. She had consumed unpasteurized milk a few weeks before the onset of her symptoms. The spinal physical examination showed impaired sensation below the knees, 4/5 strength in the lower extremities, and weak patellar tendon and Achilles tendon reflexes. Laboratory tests indicated a normal white cell count, an increased erythrocyte sedimentation rate (ESR; 50 mm/hour), and an elevated level of C-reactive protein (CRP; 91 mg/L). The interferon-γ release assay (T-spot test) was negative. The Rose–Bengal test result was positive, and the serum agglutination test showed an increased diluted titer of 1:400. *Brucella melitensis* was obtained from blood cultures. Computed tomography revealed a collapsed L3/4 intervertebral space (Figure 1A) and destruction of the L3 and L4 vertebrae (Figure 1B and C). Magnetic resonance imaging showed L3/4 spondylodiscitis (Figure 1D), a massive psoas abscess extending from L3 to the iliac fossa (Figure 1E), and an L3/4 epidural abscess (Figure 1F).

**Figure 2.**
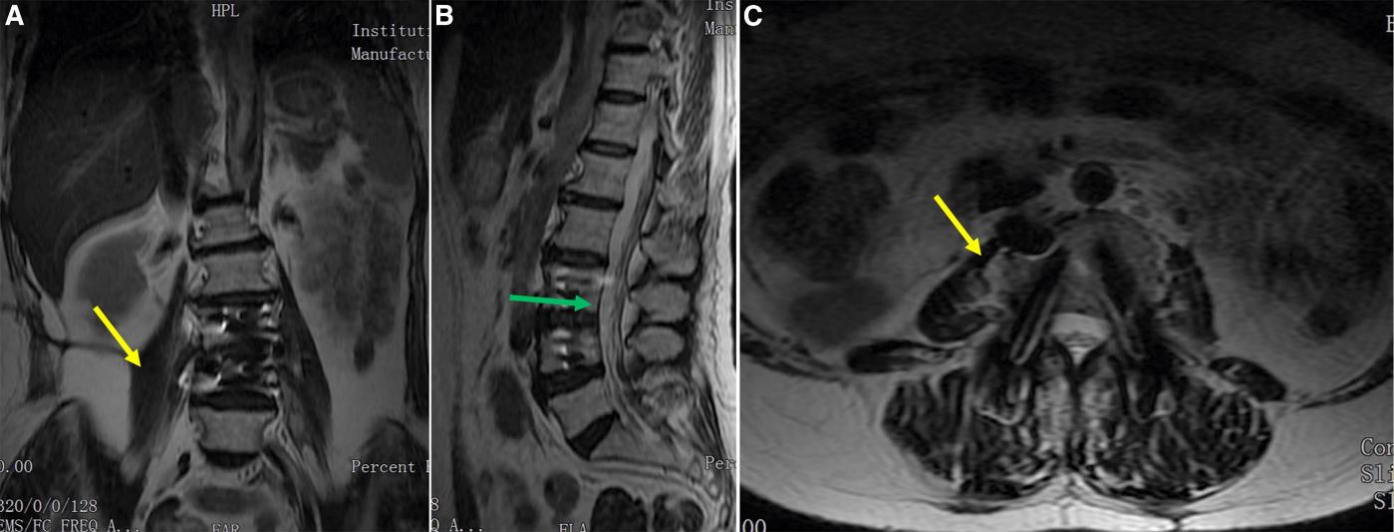
Magnetic resonance (MR) images at the 12-month follow-up. (**A–C**) Coronal, sagittal, and axial MR images indicate that the psoas abscess (yellow arrow) and epidural abscess (green arrow) are cured.

Given the intolerable back pain, massive psoas and epidural abscesses, and neurologic deficit, surgical treatment was recommended. The patient underwent a one-stage anterior debridement of the abscesses, interbody bone grafting, and internal fixation, followed by posterior debridement and pedicle screw fixation. The patient’s symptoms significantly improved postoperatively. A histopathologic examination and bacterial cultures confirmed the diagnosis of *Brucella *spondylodiscitis (BS). Anti-brucellosis chemotherapy with doxycycline at a dose of 200 mg/day and rifampicin at a dose of 600 mg/day was administered for 6 months. At the 12-month follow-up, the ESR and CRP values were normal. Magnetic resonance imaging showed that the epidural and paraspinal abscesses were cured (Figure 2).

*Brucella *spondylodiscitis most frequently affects the lumbar spine (81.2%),^1^ but BS with massive psoas and epidural abscesses is a rare condition. Treatments for BS include medication and surgery.^2^ Surgical indications include progressive deformity, large abscess, neurological deficit, and intolerable pain.^3^ After surgery, regular anti-brucellosis chemotherapy and close follow-up are recommended to prevent relapses.
